# *Vaccinium fangianum* (Ericaceae), a new species from Northwest Yunnan, China, with critical notes on *V. ardisioides*

**DOI:** 10.3897/phytokeys.275.197108

**Published:** 2026-05-20

**Authors:** Xiao-Han Liang, Jing-Bo Ni, Yi-Hua Tong

**Affiliations:** 1 State Key Laboratory of Plant Diversity and Specialty Crops & Laboratory of Plant Resources Conservation and Sustainable Utilization, South China Botanical Garden, Chinese Academy of Sciences, Guangzhou, Guangdong, 510650, China State Key Laboratory of Plant Diversity and Specialty Crops & Laboratory of Plant Resources Conservation and Sustainable Utilization, South China Botanical Garden, Chinese Academy of Sciences Guangzhou China; 2 University of Chinese Academy of Sciences, Beijing, 100049, China University of Chinese Academy of Sciences Beijing China; 3 South China National Botanical Garden, Chinese Academy of Sciences, Guangzhou, Guangdong, 510650, China South China National Botanical Garden, Chinese Academy of Sciences Guangzhou China

**Keywords:** Name misapplication, new synonym, taxonomy, Vaccinieae, *Vaccinium* sect. *Epigynium*

## Abstract

*Vaccinium fangianum*, a new species of Ericaceae from Yunnan Province, China, is described and illustrated. This species was previously misidentified as *Vaccinium ardisioides*. Morphologically, the new species is similar to *Vaccinium ardisioides* in its epiphytic habit, pseudo-whorled leaves with entire blade margins, and axillary racemose inflorescences but differs in having wider leaf blades, a deeply lobed calyx limb, a yellowish-green corolla that is villous internally, and spurless anthers with tubules ca. 1.7× as long as thecae. Detailed descriptions, analytical photographic plates, conservation status information, and a distribution map of the two species are also provided. In addition, *V. rubescens* was confirmed to be a synonym of *V. ardisioides* in this study.

## Introduction

The genus *Vaccinium* L. (Ericaceae), with approximately 485 species distributed worldwide, is the largest genus in the tribe Vaccinieae (Vander Kloet and Dickinson 2009; POWO 2026). As one of the world’s most biodiverse countries, China currently has recorded 105 species of *Vaccinium*, including the recently described *Vaccinium dehongense* Y.H.Tong and *Vaccinium longisetosum* X.C.Xie & Y.H.Tan (Tong et al. 2024; Xie et al. 2025; POWO 2026). Yunnan Province harbors approximately 50 species of *Vaccinium*, representing the highest diversity of this genus in China (Fang 1991; Huang and Fang 1991; Fang and Stevens 2005; Guo et al. 2023; Tong et al. 2024). When examining the *Vaccinium* specimens collected from Yunnan, it was found that the application of two names, viz., *Vaccinium ardisioides* Hook.f. ex C.B.Clarke and *Vaccinium rubescens* R.C.Fang, was problematic.

**Figure 1.**
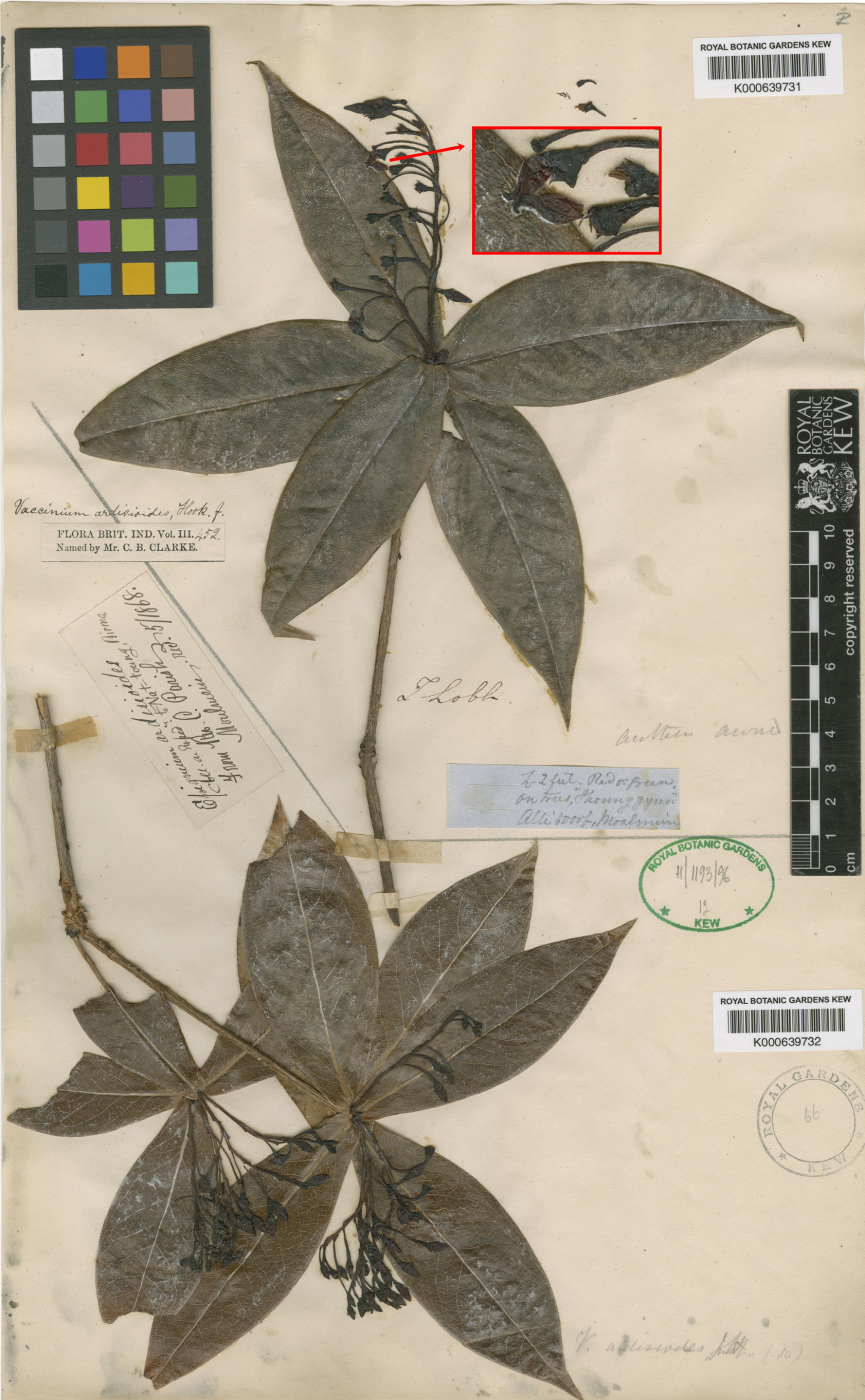
Syntypes of *Vaccinium ardisioides* (*T. Lobb s.n*., K, K000639731 & *C. Parish s.n*., K, K000639732), with the inserted image showing the flower with a torn corolla and short corolla lobes (© copyright Royal Botanic Gardens, Kew).

*Vaccinium ardisioides* was described by Clarke (1882) based on two collections from Myanmar (*T. Lobb s.n*. & *C. Parish s.n*.). Huang and Fang (1991) reported its distribution in Southwest Yunnan, China, and discussed that the materials from Southwest Yunnan matched Clarke’s description in overall morphology, such as pseudowhorled leaves with oblong-ovate blades and entire blade margins, racemose inflorescences, clavate pedicels, shortly campanulate calyx limbs, and spurred anthers, but differed slightly in inflorescence length (7–12 cm in Chinese materials vs. 5–7.5 cm in the protologue), pedicel length (ca. 1.5 cm in Chinese materials vs. 0.6–1.3 cm in the protologue), and the degree of corolla division (shallowly lobed in Chinese materials vs. deeply lobed in the protologue). Fang may not have seen the type collections at that time, as both type collections bear young inflorescences with unopened flowers (Fig. 1), which is exactly the reason for the discrepancies in inflorescence and pedicel length. However, it is unclear where Clarke obtained the information on the deeply lobed corolla, since almost all flowers on the type specimens are still young and unopened, except one with a torn corolla and short corolla lobes. According to the examination of many specimens of this species (see the text below), it truly has the same shallowly lobed corolla as Fang observed.

**Figure 2.**
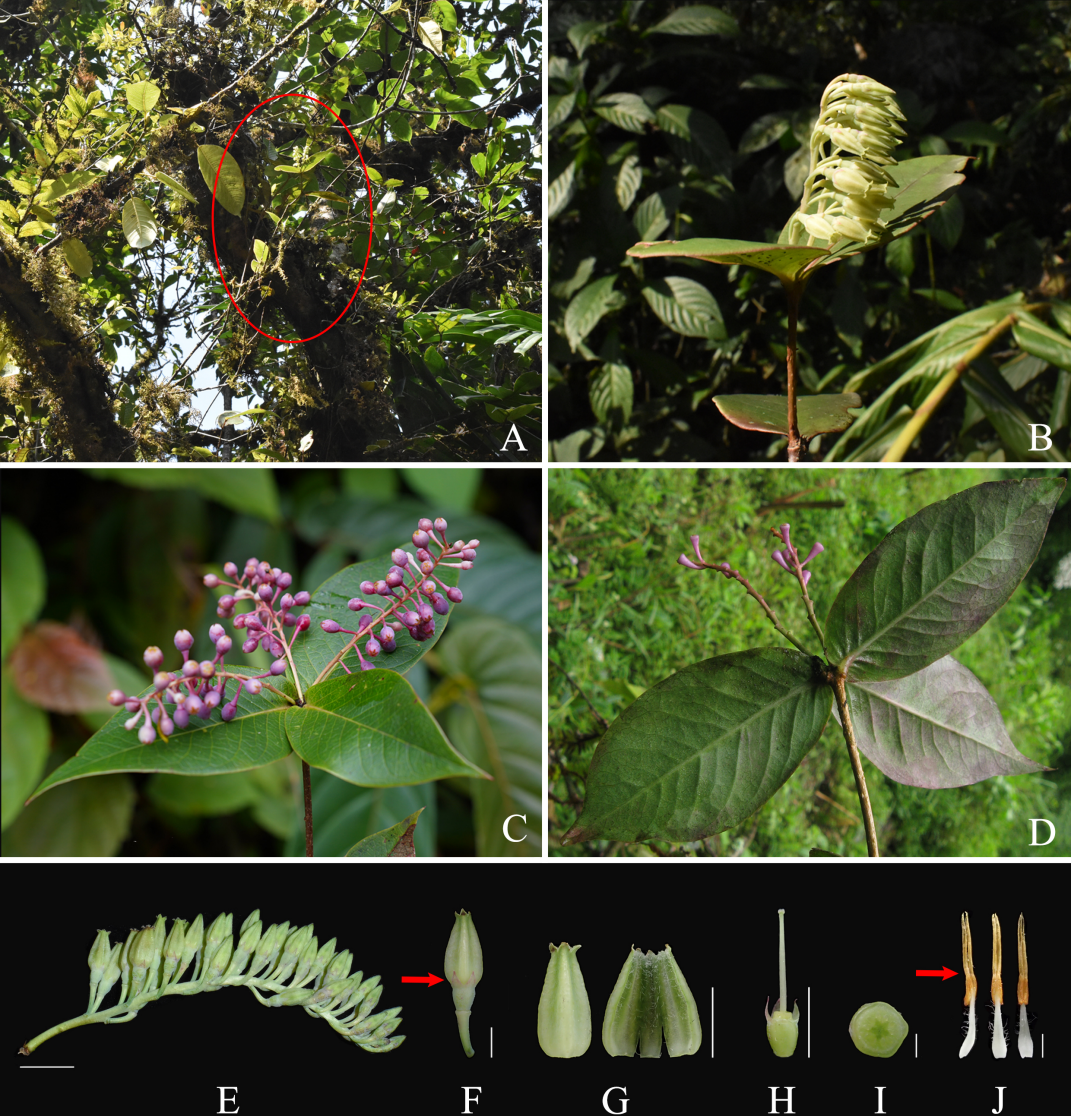
*Vaccinium fangianum* sp. nov. **A**. Habitat, the red oval indicating this species epiphytic on a tree; **B**. Flowering branch; **C**. Fruiting branch; **D**. Fruiting branch, with fruits fallen off and persistent fruit pedicels; **E**. Inflorescence; **F**. Flower, the red arrow indicating the deeply lobed calyx limb; **G**. Corolla, external (left) and internal (right) view; **H**. Calyx, disc, and style with front two calyx lobes removed, showing the deeply lobed calyx limb; **I**. Ovary cross-section, showing pseudo-10 locules; **J**. Stamens, lateral (left), abaxial (middle), and adaxial (right) view, the red arrow indicating the spurless anther. Scale bars: 1 cm (**A**); 5 mm (**F–H**); 1 mm (**I–J**). All photos by Y.H.Tong except (**C**) by Zi Wang (**A–B, E–J** based on holotype, (**C**) not vouchered, (**D**) based on *Y. H. Tong 11071408*).

**Figure 3.**
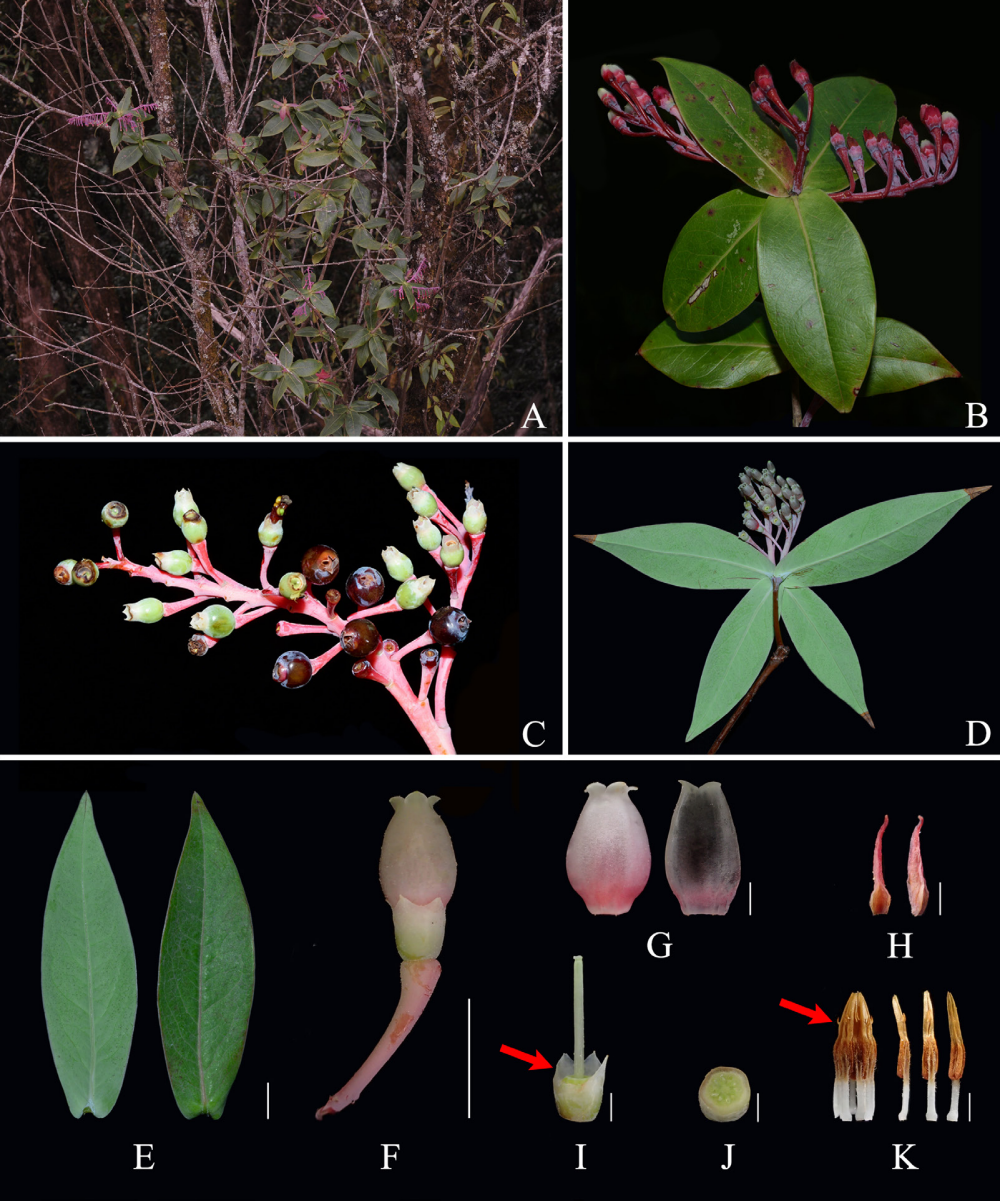
*Vaccinium ardisioides***A**. Habitat; **B**. Flowering branch; **C**. Infructescences; **D**. Flowering branch, showing petioles and abaxial surface of leaves; **E**. Leaf blades, abaxial (left) and adaxial (right) view; **F**. Flower; **G**. Corolla, external (left) and internal (right) view; **H**. Bracteole, adaxial (left) and lateral (right) view; **I**. Calyx tube, disc, and style with front two calyx lobes removed, the red arrow indicating the shallowly lobed calyx limb; **J**. Ovary cross-section, showing pseudo-10-locular ovary; **K**. Androecium and stamens, lateral (left), abaxial (middle), and adaxial (right) view, the red arrow indicating the spurred anthers. Scale bars: 1 cm (**E–F**); 2 mm (**G, I–K**); 0.5 mm (**H**). Photos (**A**) by B. Liu; (**B**) by C. Liu; (**C**) by Y. Yang; **D–K** by Y.H.Tong (**A–C** not vouchered; **D–K** based on *J. Q. Zhu TYH-2566*).

However, Fang (1999) curiously described a new species, i.e., *V. rubescens* R.C.Fang, several years later based on the same materials from Southwest Yunnan. She inexplicably compared her new species with a very strange “*V. ardisioides*,” which has ovate or elliptic leaf blades, slender pedicels, white-green and urceolate-tubular corollas, subglabrous filaments, and spurless anthers. Until the description of *V. ardisioides* in the account of *Vaccinium* in “Flora of China” (Fang and Stevens 2005) was checked, it was not clear that the strange “*V. ardisioides*” recognized by Fang was from Northwest Yunnan (Dulongjiang). After examination of those specimens identified as *V. ardisioides* by Fang from Dulongjiang, it was found that the key characters of these materials matched well with those of the strange “*V. ardisioides*” but were clearly distinct from the true *V. ardisioides*, such as the leaf blade shape (ovate-lanceolate to ovate vs. elliptic to oblong-lanceolate; Fig. 2D vs. Fig. 3E), the degree of calyx limb lobation (lobed more than 1/2 vs. lobed less than 1/2; Fig. 2H vs. Fig. 3I), calyx lobe shape and length (narrowly triangular, ca. 1.5 mm long vs. widely triangular, 0.5–1 mm long; Fig. 2F vs. Fig. 3F), corolla color (yellowish green vs. white-pinkish or pinkish; Fig. 2G vs. Fig. 3G), indumentum on the internal corolla surface (villous vs. glabrous; Fig. 2G vs. Fig. 3G), the absence or presence of anther spurs (absent vs. present; Fig. 2J vs. Fig. 3K), and the relative length of tubules and thecae (tubules ca. 1.7× as long as thecae vs. tubules as long as thecae; Fig. 2J vs. Fig. 3K). A more detailed morphological comparison between the two species is presented in Table 1.

**Table 1.** Morphological comparison of *Vaccinium fangianum* and *V. ardisioides*. Data for the latter species were obtained from Fang (1991), Watthana (2015), and the examined specimens listed in the text.

**Characters**	***Vaccinium fangianum***	***Vaccinium ardisioides***
Leaf blade shape	Ovate-lanceolate to ovate, length:width 1.8–2.6, widest near the base	Elliptic to oblong-lanceolate, length:width 3–3.5, widest at the middle
Pairs of lateral veins	8–12	10–16
Calyx limb lobation	Lobed more than 1/2	Lobed less than 1/2
Calyx lobes	Narrowly triangular, ca. 1.5 mm long, without apical gland	Widely triangular, 0.5–1 mm long, with apical gland
Corolla	Yellowish green, tubular, villous internally	White-pinkish or pinkish, tubular to urceolate, glabrous internally
Anther tubules	ca. 1.7× as long as thecae, without spurs on the back	As long as thecae, with two short spurs at the middle on the back.

Perhaps because the inflorescences and pedicels of the species from Northwest Yunnan are slightly shorter than those of the species from Southwest Yunnan (the true *V. ardisioides*) and better match the protologue of *V. ardisioides*, Fang misapplied the name *V. ardisioides* to the collections from Northwest Yunnan and published another redundant name (*V. rubescens*) for the species from Southwest Yunnan (the true *V. ardisioides*). Thus, it was concluded that the materials from Northwest Yunnan (Dulongjiang) represent an undescribed species distinct from *V. ardisioides*, while *V. rubescens* is conspecific with *V. ardisioides*. As the descriptions of *V. ardisioides* in the protologue and the account of *Vaccinium* in Flora of China (Fang and Stevens 2005) are both inaccurate, a new description of this species is provided here. The new species from Dulongjiang is also described and illustrated below.

## Materials and methods

Flowering and fruiting materials were collected from Yunnan during three field trips in July 2011, April 2022, and March 2023, respectively. Herbarium specimens or specimen photos (including types) from E, HITBC, IBSC, K, KUN, P, PE, and SZ (herbarium acronyms follow Thiers 2026, updated continuously) and JSTOR (available at https://www.jstor.org/) and GBIF (available at https://www.gbif.org/zh/) were examined. Lists of examined specimens are provided in the text below. Descriptions were based on both living and dried collections. Measurements were performed with a ruler, and small plant parts were observed and measured under a stereomicroscope (Mshot-MZ101, Guangzhou Micro-shot Technology Co., Ltd., Guangzhou, China). Terminology follows that of Fang and Stevens (2005), Beentje (2016), and Nuraliev et al. (2020). The collections are arranged geographically (from NW to SE) and then chronologically in the list of examined specimens.

## Taxonomic treatment

### Vaccinium ardisioides

Taxon classification

Plantae

EricalesEricaceae

Hook. f. ex C. B. Clarke, Fl. Brit. India 3(9): 452 (1882)

CDE34226-FF38-5C3D-B78E-833F6DAE72E7

Fig. 3

= Vaccinium rubescens R. C. Fang, Novon 9(2): 174 (1999); R. C. Fang & P. F. Stevens, Fl. China 14: 494 (2005). syn. nov. Type: **China**. Yunnan Province • Ximeng County, 2000 m a.s.l., *Y. C. Du D580156* [fl.] (holotype: KUN, 1209485!; isotype: KUN, 1209486!).

#### Type.

**Myanmar**. [Bago Region?] • Thoung-gyun [Taungoo], 1800 m a.s.l., *T. Lobb s.n*. [young fl.] (Lectotype, K, K000639731, image!, designated by Sleumer 1941: 477); remaining syntype: MYANMAR. [Kayin State] • Nat-toung, *C. Parish s.n*. [young fl.] (K, K000639732, image!).

#### Description.

Evergreen ***shrubs***, epiphytic, 1–2 m tall. ***Twigs*** reddish brown when young, becoming grayish brown when older, terete, glabrous, without or with very sparse lenticels. ***Buds*** ovoid-conical, ca. 7 mm long, ca. 3.5 mm wide; bud scales ca. 5 mm long, triangular, apex acuminate, glabrous, caducous. ***Leaves*** alternate, pseudowhorled, 3–8 per whorl. ***Petiole*** subsessile, light green, glabrous and pruinose. ***Leaf blades*** leathery, elliptic to oblong-lanceolate, 7.5–13 × 2.5–4 cm, length:width 3–3.5, base narrowly obtuse or subcordate, margin entire, apex acuminate to shortly caudate, glabrous on both sides; midvein raised on both sides, more so abaxially; lateral veins 10–16 pairs, anastomosing near the margin, raised on both sides; veinlets conspicuous and raised on both sides. ***Inflorescences*** racemose, axillary, (10) 17–27-flowered; rachis 6–11 cm long, pink or purplish red, rarely greenish, angular, pruinose, flowers secund; bracts lanceolate, ca. 2.5 mm long, caducous; bracteoles linear-lanceolate, ca. 1.5 mm long, inserted at the base of pedicel, caducous; pedicels expanded upwards, clavate to obconical, 0.7–1.5 cm long, pink or purplish red, rarely greenish, pruinose, articulated with calyx. ***Calyx*** tube cupulate, 1.7–2.5 mm long, purplish red or purple, rarely greenish, glabrous and pruinose; calyx limb shortly campanulate, ca. 2.2 mm long, pink or purplish red, rarely yellowish green, lobed less than 1/2 to base; lobes broadly triangular, apex acute, with a terminal gland, 0.5–1 mm long. ***Corolla*** white-pinkish or pinkish, turning to purplish red later; tubular-urceolate, 7–9 mm long, glabrous externally and internally; lobes reflexed, broadly triangular, ca. 1 mm long, apex obtuse. ***Stamens*** 10, ca. 7.5 mm long; filaments flat, 2–3.5 mm long, lower half nearly glabrous, upper half pubescent, more so at the apex; anthers ca. 5 mm long; thecae 2–3 mm long; tubules almost as long as thecae, opening by terminal short introrse slits ca. 1 mm long at the apex, with 2 small erect spurs at the middle abaxially, spurs ca. 0.5 mm long. ***Ovary*** inferior, pseudo-10-locular, each locule with several ovules; disc ca. 3 mm in diam., glabrous; style ca. 8.5 mm long, exserted ca. 1 mm from the connate anther tubules, glabrous, slightly constricted at the joint with stigma, stigma truncate. ***Infructescence*** rachis 8–15 cm long, glabrous; fruit pedicel purplish-red, pruinose. ***Fruit*** globose, 4–6 mm in diam., young fruit yellowish green, purple-black at maturity, glabrous, pruinose, calyx lobes persistent.

#### Chinese name.

红梗越橘 (Chinese pinyin: hóng gěng yuè jú).

#### Distribution and habitat.

**Figure 4.**
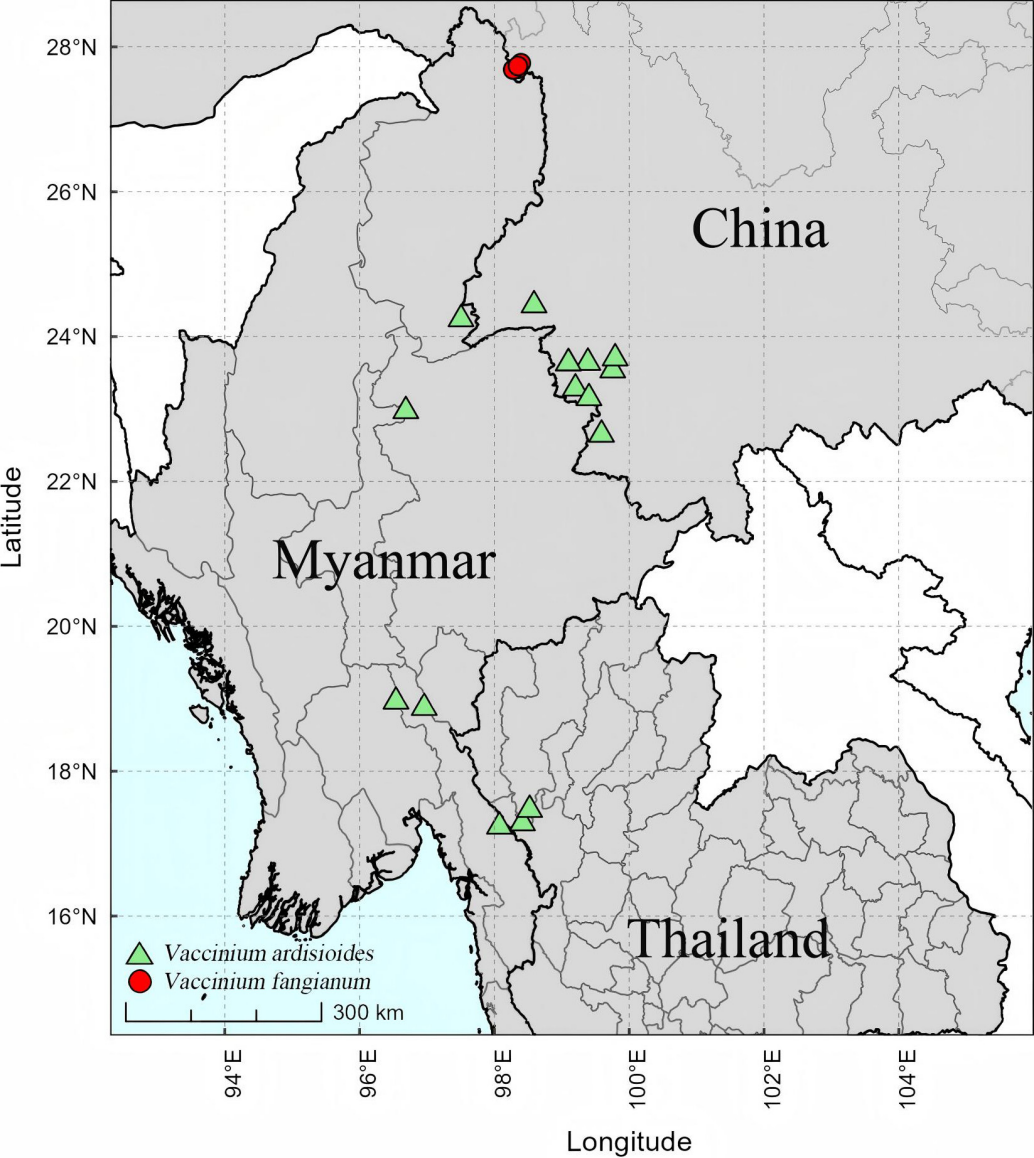
Distribution map of *Vaccinium fangianum* and *V. ardisioides*.

This species is distributed in Southwest China (Southwest Yunnan), East Myanmar (Kachin State, Kayin State, Mon State, and Shan State), and Northwest Thailand (Chiang Mai and Tak) (Sleumer 1941; Watthana 2015) (Fig. 4). This species is usually epiphytic on trees in evergreen broad-leaved forests or along riversides at elevations of 1400–2500 m.

#### Phenology.

Flowering and fruiting from February to June.

#### Conservation status.

Based on specimen records, more than 20 locations of *V. ardisioides* have been recorded, and its distribution range covers a relatively large area across China, Myanmar, and Thailand, with an expected extent of occurrence (EOO) of ca. 280,000 km^2^. This species is not economically valuable, and no evidence of severe population decline has been observed. Therefore, the conservation status of this species is assessed as Least Concern (LC) (IUCN Standards and Petitions Committee 2024).

#### Taxonomic notes.

The types, including paratypes, of *V. rubescens* are completely consistent with those of typical *V. ardisioides* in both key vegetative and reproductive characters, such as 5–7 pseudo-whorled leaves with subsessile petioles, leathery oblong-lanceolate leaf blades with narrowly obtuse bases, entire margins, and acuminate apices, midveins raised on both sides, lateral veins 10–12 pairs, anastomosing near the margin and raised on both sides, axillary racemose inflorescences with many secund flowers and purplish-red angular rachises, clavate and purplish-red pedicels, cupulate calyx tubes, triangular calyx lobes, ovoid-conical floral buds, and tubules nearly as long as thecae and with two short spurs at the middle abaxially. Therefore, *V. rubescens* should be treated as a synonym of *V. ardisioides*.

#### Additional specimens examined.

**China. Yunnan Province** • Cangyuan County, Wengding mountain pass to Mengleng reservoir, 23°17'14.02"N, 99°12'56.98"E, 2016 m a.s.l., 2 March 2014, *C. Liu, H. R. Zi & J. C. Zhao 14CS8412* [fl.] (KUN, 1338863) • Cangyuan County, Shanjia Xiang to Gaduocun, Nandan Line, 23°09'06.65"N, 99°24'03.73"E, 1996 m a.s.l., 20 March 2018, *C. Liu, J. D. Ya & C. H. Li 18CS16734* [fl.] (KUN, 1486788) • Cangyuan County, 15 April 2022, J. Q. Zhu TYH-2566 [fl.] (IBSC) • Gengma County, Xishan, 2150 m a.s.l., 4 May 1955, *P. Y. Mao 05574* [young fr.] (IBSC, 0528635; KUN, 1209488; PE, 01909132); ibid., 1 May 1964, *Y. H. Li 004983* [young fr.] (HITBC, 31659; KUN, 1209489; IBSC, 0420053) • Gengma County, Nantianmen, 23°37'37.46"N, 99°21'46.12"E, 1992 m a.s.l., 7 May 2021, *Gengma Chinese Medicine Resources Expedition 5309260805* [young fr.] (KUN, 1530538) • Lu-se [Mangshi City], 2100 m a.s.l., 5 March 1934, *H. T. Tsai 56424* [fl.] (KUN, 1209487 & 1209490; PE, 00197058; SZ, 00137852) • Shuangjiang County, Mengku Daxueshan, ancient tea garden, 2500 m a.s.l., 1 April 2005, *S. S. Zhou 2566* [fl.] (PE, 01909131) • Shuangjiang County, Chenjiabanleng Houshan, 23°34'4.27"N, 99°43'11.90"E, 2387 m a.s.l., 7 June 2020, *Shuangjiang Chinese Medicine Resources Expedition 5309250482* [young fr.] (KUN, 1531407). **Myanmar. Kachin State** • Bhamo District, Sinlum Kaba, Kachin Hills, 1524 m a.s.l., 11 April 1912, *J. H. Lace 5784* [fl.] (E, E00887329, image). **Mon State** • pass over Dawna Range, Paingkyu [Paingkyon] to Talé, 1524 m a.s.l., 23 February 1909, *J. H. LACE 4647* [fl.] (E, E00887330, image). **Thailand. Tak Province** • Doi Pae Poe, approximately 90 km. NW of Tak, 17°17'N, 98°25'E, 1400 m a.s.l., 13 March 1968, *B. Hansen & T. Smitinand 12900* [young fl.] (P, P04473481, image; E, E01580277, images).

### Vaccinium fangianum

Taxon classification

Plantae

EricalesEricaceae

X.H.Liang & Y.H.Tong sp. nov.

45F41004-C62B-5BDF-BCDC-D6CB3C8D1CC8

urn:lsid:ipni.org:names:77380492-1

Fig. 2

Vaccinium ardisioides auct. non Hook. f. ex C. B. Clarke: Fang & Stevens, Fl. China 14: 493 (2005), p. p.

#### Type.

**China**. Yunnan Province • Gongshan County, Dulongjiang Township, Maku Village, 27°40'27.79"N, 98°16'20.45"E, 1231 m a.s.l., 8 March 2023, *Y. H. Tong, J. B. Ni, B. M. Wang & W. H. Pan TYH-2595* [fl.] (holotype: IBSC; isotypes: IBSC, KUN, PE).

#### Description.

Evergreen ***shrubs***, epiphytic on tree trunks or branches, 0.5–1.2 m tall. ***Twigs*** fuscous when young, becoming grayish brown when older, terete, glabrous, with scattered white lenticels. ***Buds*** ovoid-globose, ca. 5 mm long, 3 mm wide; bud scales ca. 4 mm long, triangular, apex acute, glabrous, caducous. ***Leaves*** alternate, pseudowhorled, 2–5 per whorl. ***Petiole*** subsessile, 1–2 mm long, light green, glabrous and pruinose. ***Leaf blades*** leathery, ovate-lanceolate to ovate, 6–14 × 3–6 cm, length:width 1.8–2.6, base broadly obtuse or subcordate, margin entire, slightly revolute, apex acuminate, glabrous on both sides; midvein together with lateral veins impressed adaxially, raised abaxially; lateral veins 8–12 pairs, anastomosing near the margin; veinlets slightly conspicuous on both sides. ***Inflorescences*** racemose, axillary, (10–)24–35-flowered; rachis 5–10 cm long, greenish, angular, glabrous, slightly pruinose, flowers secund; bracts narrowly triangular, 2.5 mm long, bracteoles not seen; pedicels expanded upwards, clavate, 6–7.5 mm long, greenish, glabrous, articulated with calyx. ***Calyx*** tube cupulate, ca. 2.5 mm long, greenish, glabrous, pruinose; calyx limb 1.7–2.0 mm long, yellowish green, nearly lobed to the base; lobes narrowly triangular, apex acute, yellowish green, more or less tinged with purplish, ca. 1.5 mm long. ***Corolla*** yellowish green, tubular, 5-angled, 7–9 mm long, glabrous externally, villous internally; lobes triangular, ca. 1 mm long, spreading or slightly reflexed. ***Stamens*** 10, 6.6–7 mm long, filaments flat, ca. 3 mm long, pilose; anthers coherent, ca. 4 mm long, without spurs abaxially; thecae ca. 1.5 mm long; tubules ca. 2.5 mm long, opening by terminal short introrse slits ca. 1 mm long. ***Ovary*** inferior, pseudo-10-locular, each locule with several ovules; disc ca. 2.5 mm in diam., glabrous; style ca. 7.5 mm long, exserted ca. 1 mm from the connate anther tubules, glabrous, stigma slightly expanded, capitate. ***Infructescence*** rachis 5–12 cm long, glabrous. ***Fruit pedicel*** pale purplish, carnosus. ***Fruit*** ellipsoid, ca. 3 mm in diam., young fruit yellowish green, turning purplish red later and finally white tinged with purple at maturity, glabrous, calyx lobes persistent.

#### Distribution and habitat.

*Vaccinium fangianum* is currently known only from Dulongjiang Township, Gongshan County, Yunnan Province, Southwest China (Fig. 4). It is usually epiphytic on tree trunks in evergreen broad-leaved and mixed forests at elevations of 1200–1500 m.

#### Phenology.

Flowering from March to April and fruiting in May to July.

#### Etymology.

The species is named in honor of Prof. Rhui-Cheng Fang, who has made great contributions to our knowledge of *Vaccinium* in China. The Chinese name is given as 瑞征越橘 (Chinese pinyin: ruì zhēng yuè jú).

#### Conservation status.

Although this species is known only from Dulongjiang Township, its type locality is very close to the China–Myanmar border, so it is likely also distributed in adjacent Northeastern Myanmar. Therefore, until further investigations are conducted, this species should be classified as Data Deficient (DD) according to the IUCN Red List conservation categories (IUCN Standards and Petitions Committee 2024).

#### Taxonomic notes.

*Vaccinium fangianum* should be assigned to *V.* sect. *Epigynium* (Klotzsch) Hook.f. due to its evergreen habit, pseudo-verticillate leaves, elongated racemose inflorescences, and stamens without spurs on the back of anthers (Sleumer 1941; Stevens 1969; Vander Kloet and Dickinson 2009). Notably, species of *V.* sect. *Epigynium* usually have serrate leaf margins, but *V. fangianum* and *V. ardisioides* uniquely bear entire leaf blades, which can distinguish them from other congeners in this section (Fang and Stevens 2005). In the key to *Vaccinium* in the account of the “Flora of China” (Fang and Stevens 2005), *V. fangianum* is keyed out as close to *V. ardisioides*. The main differences between the two species have been indicated in the Introduction and Table 1. Furthermore, they have an allopatric distribution: *V. fangianum* is endemic to Northwest Yunnan (Dulongjiang), while *V. ardisioides* is distributed in Southwest Yunnan, China, Southeast Myanmar, and West Thailand.

#### Additional specimens examined (paratypes).

**China. Yunnan Province** • Nujiang Lisu Autonomous Prefecture, Gongshan County, Dulongjiang Township: The Fourth Village on the West Bank of Dulong River, Maku, 1300 m a.s.l., 19 November 1959, *G. M. Feng 24370* [st.] (KUN, 0231235, 0231236 & 0231237) • Mabiluo, 1310 m a.s.l., 30 December 1990, *Dulongjiang Expedition 1395* [fl. bud] (KUN, 0231242; CAS, 519293, image) • Telawang River, 1380 m a.s.l., 31 January 1991, *Dulongjiang Expedition 3847* [fl. bud] (KUN, 0231243 & 0231244), *3876* [young fl.] (KUN, 0231245 & 0231246; CAS, 519294, image) • Mabidang, 1400 m a.s.l., 8 March 1991, *Dulongjiang Expedition 4626* [fl.] (KUN, 0231238 & 0231239) • Bapo, 1380 m a.s.l., 9 March 1991, *Dulongjiang Expedition 4676* [fl.] (KUN, 0231249 & 0231250); ibid., 1350 m a.s.l., 15 May 1991, *Dulongjiang Expedition 6759* [young fr.] (KUN, 0231240 & 0231241) • Qinlangdang, 1280 m a.s.l., 10 March 1991, *Dulongjiang Expedition 4485* [fl.] (KUN, 0231248; CAS, 288443, image) • Silaluo, 1450 m a.s.l., 13 March 1991, *Dulongjiang Expedition 4725* [fl.] (KUN, 0231247) • Mengluo, 1320 m a.s.l., 14 May 1991, *Dulongjiang Expedition 6727* [young fr.] (KUN, 0231251 & 0231252) • Kongdang to Bapo, 27°49'3.53"N, 98°19'22.32"E, 1448 m a.s.l., 13 July 2011, *Y. H. Tong 11071311* & *11071312* [st.] (IBSC) • Mengdang to Langwangduo, 27°42'37.25"N, 98°21'17.11"E, 1414 m a.s.l., 14 July 2011, *Y. H. Tong 11071408 & 11071409* [st.] (IBSC).

## Supplementary Material

XML Treatment for Vaccinium ardisioides

XML Treatment for Vaccinium fangianum

## Additional information

### Artificial Intelligence (AI) use

The authors accept full responsibility for the content of the manuscript, including the disclosure of any use of AI.

No AI tools were used in the preparation of this manuscript.

### Author ORCIDs

X-H. Liang https://orcid.org/0009-0000-6657-489X

J-B. Ni https://orcid.org/0000-0002-1695-6141

Y-H. Tong https://orcid.org/0000-0002-5034-005X

### Data availability

All of the data that support the findings of this study are available in the main text.
